# Building the Field with Harmonized and Context-Sensitive Measurement in Mental Health

**DOI:** 10.1192/j.eurpsy.2026.10962

**Published:** 2026-07-31

**Authors:** K. R. Krause, C. Gosling, S. Monga, G. Rada, N. Armstrong, A. Chevance, G. Zareen

**Affiliations:** 1 Centre for Research in Epidemiology and Statistics (CRESS-UMR1153), Université Paris Cité; 2 DysCo Laboratory, Université Paris Nanterre, Paris, France; 3 Department of Psychiatry, University of Toronto, Toronto, Canada; 4 Epistemonikos Foundation, Santiago de Chile, Chile; 5 Department of Anthropology and Sociology, SOAS University of London, London, United Kingdom; 6 Centre for Research in Epidemiology and Statistics (CRESS-UMR1153), Assistance Publique-Hôpitaux de Paris; 7 Inserm, CRESS – Centre de Recherche Épidémiologie et StatistiqueS, Paris, France

## Abstract

**Introduction:**

Progress in mental health is held back by shortcomings in data consistency and interpretability. At the policy level, only 31% of WHO member states reported mental-health-specific data in 2020. In research, more than 4,200 outcome measures are in use. Fewer than half of depression studies report outcomes that can be aggregated. Such fragmentation limits the comparability of trials or epidemiological surveys and causes research waste. To address this, major funders and journals launched the Common Measures in Mental Health Science (CMMHS) Initiative, which recommends four instruments to assess common symptoms and functional impairment (PHQ-9, GAD-7, RCADS-25, WHODAS 2.0).

**Objectives:**

Today, there is no perfect measure, and no measure represents a biological or diagnostic gold standard. To make common measures useful and ethical, we need to understand how well they perform across populations and contexts, and ensure that their psychometric evidence is synthesized and regularly updated. The Wellcome Trust, as a CMMHS funding member, has commissioned a project to deliver living systematic reviews of the four measures and a free, interactive platform providing up-to-date evidence on validity, reliability, and acceptability. This session will outline the project.

**Methods:**

Reviews will follow established methodological and reporting standards (COSMIN, Cochrane). Platform design will be informed by mixed-methods research with target users to promote uptake. Experts with lived experience will be integrated on advisory boards, in the co-production of review protocols, and in reflections on the impacts of measurement. The platform architecture will embed tools for literature search, screening, and data extraction as part of the backend infrastructure, ensuring these processes can be repeated efficiently to sustain the living mode.

**Results:**

The project will produce regularly updated evidence syntheses of the psychometric properties of the PHQ-9, GAD-7, RCADS-25, and WHODAS 2.0. These will be made available on an open-access platform that enables tailored queries by target use context, population, or setting. The platform will also share knowledge on the wider impacts of outcome measurement, including how instruments are experienced by those with lived experience.

**Conclusions:**

This project is guided by four principles: inclusivity, scientific excellence, user-centred design, and modular, scalable technology. The project seeks to build shared ownership across people with lived experience and professionals, including those from low- and middle-income countries. The four recommended measures should be seen as a pragmatic starting point, chosen not to privilege particular scales but to ensure researchers begin from a shared set of outcomes. The aim is to encourage uptake while also promoting *wise use*, defined as an application that is transparent about both the strengths and limitations of each measure in specific contexts.

**Disclosure of Interest:**

None Declared

